# Retroperitoneal bronchogenic cyst masquerading as an adrenal incidentaloma

**DOI:** 10.1530/EDM-25-0012

**Published:** 2025-09-12

**Authors:** Trevor Tam, Nishani Jayatunge, Louis Saada, Srinivasan Ramalingam, David Manson-Bahr, Mark Rochester, Rupa Ahluwalia, Richard Ball

**Affiliations:** ^1^ Norfolk and Norwich University Hospital, Norwich, UK; ^2^ University of East Anglia, Norwich, UK; ^3^ James Paget University Hospital, Great Yarmouth, UK

**Keywords:** adrenal, rare diseases/syndromes

## Abstract

**Summary:**

Bronchogenic cysts, developmental malformations of the primitive foregut, extremely rarely occur in the retroperitoneum. Here, we present a retroperitoneal bronchogenic cyst presenting as an adrenal incidentaloma and masquerading clinically as a phaeochromocytoma.

**Learning points:**

- Retroperitoneal bronchogenic cysts are exceptionally rare, particularly presenting as adrenal incidentalomas (AI), and can mimic other conditions such as non-functioning phaeochromocytomas.
- This case emphasises the need to consider rare conditions such as retroperitoneal bronchogenic cysts in the differential diagnosis of AI, especially when imaging findings are unusual despite normal hormonal studies. Atypical adrenal masses still warrant surgical evaluation and histopathological analysis to confirm the diagnosis and exclude rare entities.
- The occurrence of retroperitoneal bronchogenic cysts highlights the importance of understanding embryogenesis, particularly the migration pathways and foregut development.
- Histopathological examination remains crucial for diagnosing bronchogenic cysts, as they demonstrate the characteristic ciliated columnar epithelium with subepithelial smooth muscle, seromucinous glands, and cartilage.

## Background

Bronchogenic cysts are developmental abnormalities, which arise as an abnormal budding from the primitive foregut between the third and seventh weeks of embryogenesis (1). They usually occur within the mediastinum but can be found anywhere along the developmental pathway of the foregut. The location of such cysts corresponds to the stage of embryogenesis. They are seen along the tracheobronchial tree if the anomaly occurs during early stages of development, and within the lung if it happens later (2).

Bronchogenic cysts in the retroperitoneum are extremely rare, occurring because the thoracic and abdominal cavities are linked by the pericardioperitoneal canal. When these cavities are later separated by the diaphragm, a part of the tracheobronchial tree can remain and migrate, giving rise to the retroperitoneal location (3).

## Case presentation

Here we present a case of a woman in her early 30s with a non-functioning 7.5 cm cystic left adrenal mass.

Her initial symptoms were altered bowel habits, rectal bleeding, and significant weight loss within a year. She also complained of irregular menses, palpitations, and sweating. She was on treatment for fibromyalgia, and there was no significant family history.

On examination, her weight was 64.2 kg with a BMI of 23.4. Her vital signs were normal, and her abdominal examination did not reveal any mass lesion.

## Investigation

Her electrolytes, renal function tests, and thyroid function tests were all within normal limits.

Aldosterone was 110 pmol/L (normal: <750 pmol/L). Twenty-four-hour urine free cortisol, measured twice, was 12–69 nmol (normal: <130 nmol/24 h). Overnight dexamethasone suppression test showed cortisol well suppressed, <28 nmol/L. Testosterone was <0.4 nmol/L (normal: 0.3–1.7 nmol/L), DHEAS 1.3 μmol/L (normal: 0.7–11.5 μmol/L). Plasma metanephrine and urinary metanephrines were all within normal limits.

Faecal immunochemical test was positive, and faecal calprotectin was increased. Colonoscopy did not reveal any significant pathology.

**Figure 1:**
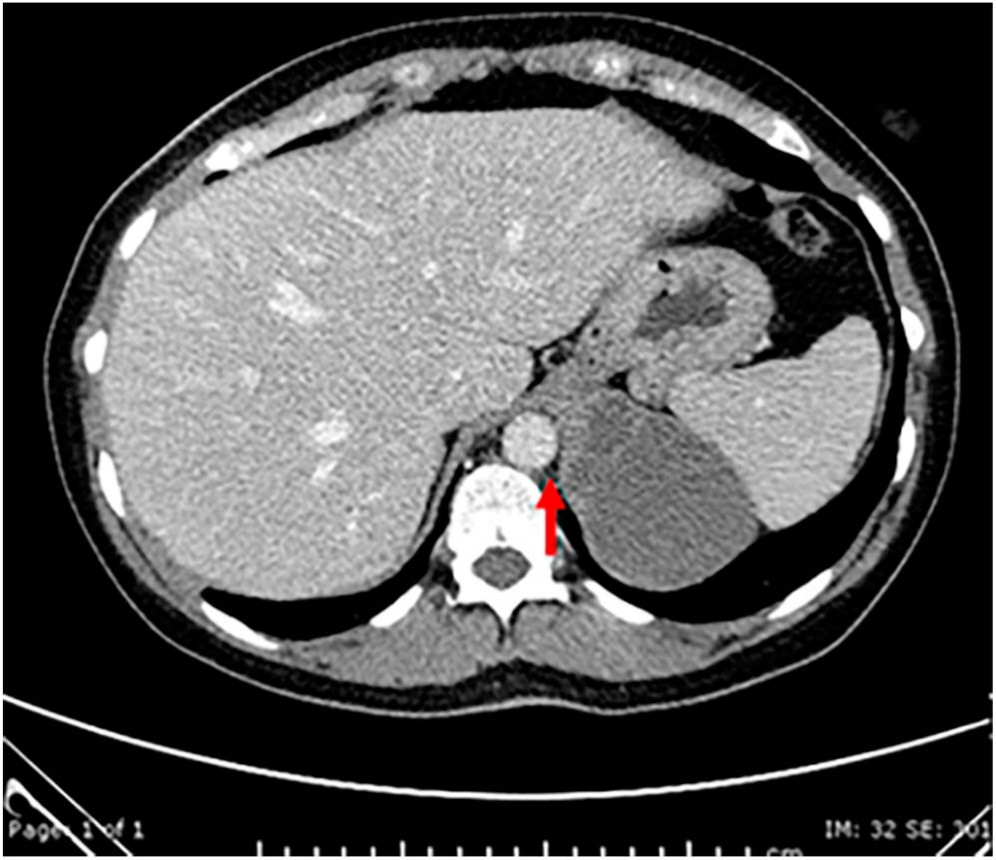
Axial 60 s post-contrast image.

**Figure 2:**
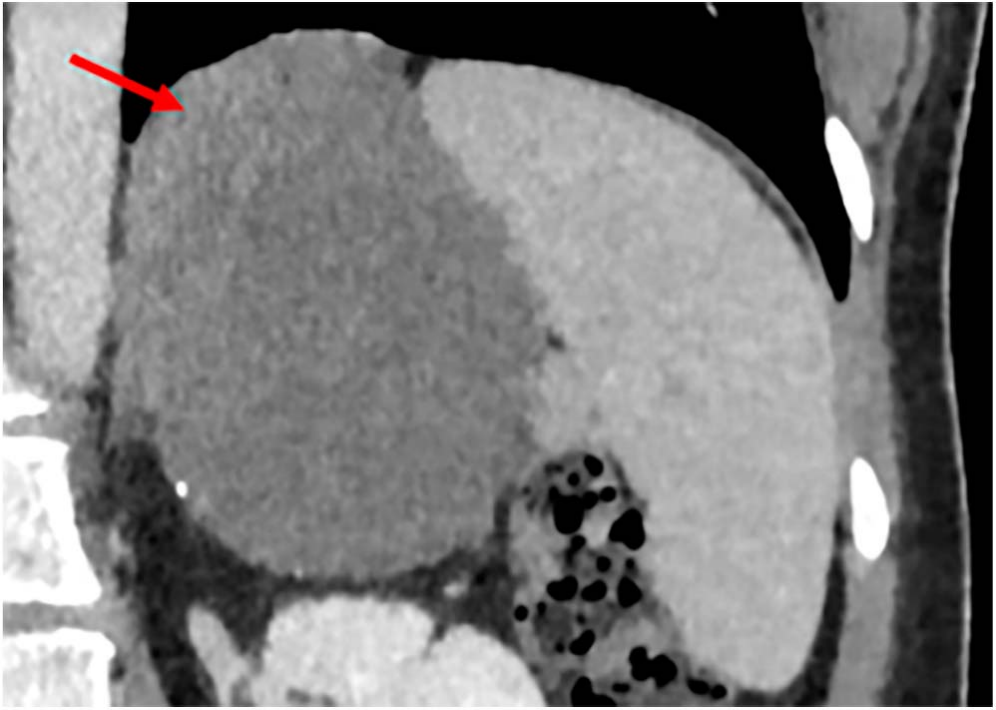
Coronal 60 s post-contrast image. There is a 7 × 6 cm multiloculated cystic retroperitoneal mass within the expected position of the left adrenal gland. The arrows point to a focus of soft tissue attenuation material at the superomedial aspect of the cystic mass, inseparable from the left diaphragmatic crus and the presumed left adrenal tissue.

A CT scan with contrast revealed a 7.5 cm, predominantly cystic lesion in the left adrenal gland (Figs 1 and 2). The superomedial subdiaphragmatic component was solid and contrast-enhancing. There was no calcification within the solid component, but there were several foci of mural calcification inferiorly. No invasion of adjacent structures was seen. The large size and the atypical features were suspicious for a non-benign aetiology.

Given the indeterminate nature of the lesion in the absence of a functioning adrenal tumour, diagnostic uncertainty was raised. In the context of these findings in a young patient, with lack of prior imaging for comparison, surgical intervention was opted for following discussion at our local adrenal multidisciplinary team meeting.

## Treatment

A left robotic adrenalectomy was undertaken. Intraoperatively, the adrenal gland appeared normal. The cystic mass was quite adherent to the crus of the diaphragm. The mass, including the remaining sac of the cyst, was completely removed. The patient made a good recovery.

Gross examination of the specimen revealed an adrenal gland (11 × 3.5 × 0.5 cm) with a completely separate cystic tumour (8 × 3.5 × 1.5 cm), together weighing 54 g. The cyst, which was multilocular and had a maximum wall thickness of 3 mm, showed evidence of rupture and appeared empty. There were no suspect areas.

**Figure 3:**
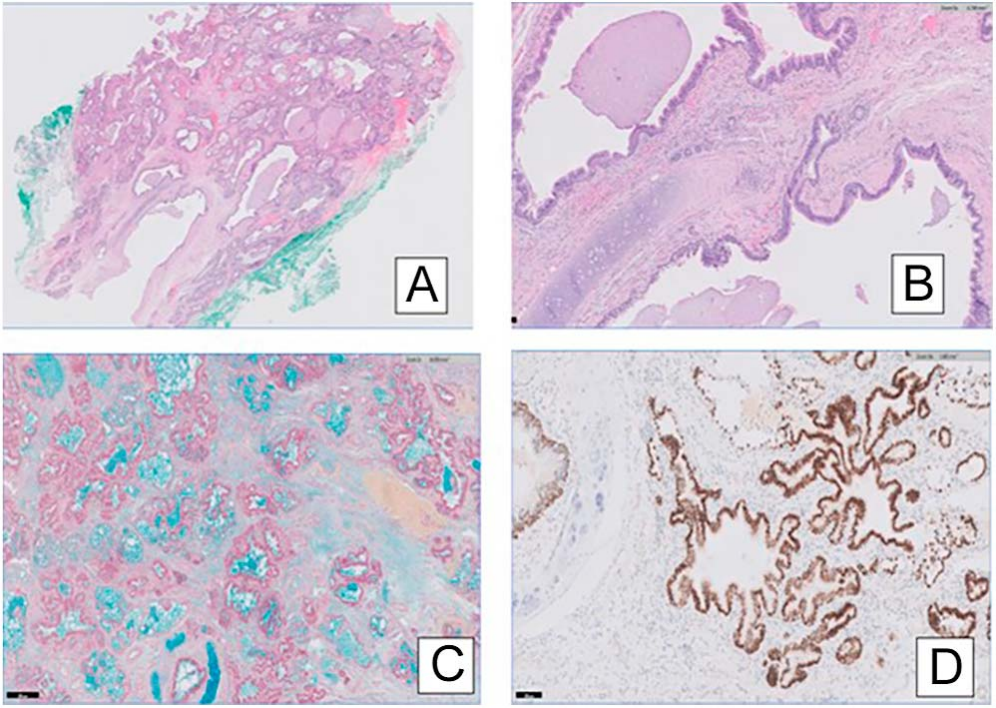
(A) This shows the multilocular nature of the cyst. (B) The sub-epithelium shows seromucinous glands and cartilage. (C) The cyst contains mucin (positive for Alcian blue). (D) The cyst epithelium is positive for thyroid transcription factor 1 (TTF1).

Microscopically, the cyst was lined by a ciliated columnar epithelium resembling respiratory tract epithelium. The subepithelial tissue contained smooth muscle bundles, seromucinous glands, and cartilage (Fig. 3).

The cyst was lined by a ciliated columnar epithelium, which was immunoreactive for cytokeratin 7 (CK7), Napsin A, and thyroid transcription factor 1 (TTF1). It did not stain for cytokeratin 20 (CK20). It contained alcianophilic, periodic acid–Schiff (PAS)-positive mucins (Fig. 3).

The pathological diagnosis was bronchogenic cyst.

## Outcome and follow-up

She was followed up in the Endocrinology Clinic, with no evidence of post-operative hypocortisolaemia. The post-operative ultrasound scan after 11 months was normal.

## Discussion

Bronchogenic cysts are a rare congenital malformation seen in 1 in 42–68 × 10^3^ individuals (4).

Among the 82 reported cases of retroperitoneal bronchogenic cyst (5), there was no significant difference between genders (41 males, 41 females), and most (64/82; 78%) were aged 21–60 years. The cysts were commonly found in the left adrenal gland (72/82; 88%). Also, 43 out of 82 patients (53%) were asymptomatic. Among patients who had symptoms, most (28/39; 72%) complained of abdominal pain.

The origin of bronchogenic cysts is believed to be from an accessory lung bud or accessory bronchus forming as an outpouching of the primitive foregut, usually associated with the tracheobronchial tree or the oesophagus. These two structures lie in a mass of loose connective tissue stretching from the root of the neck superiorly to the diaphragm inferiorly, which later becomes the mediastinum. Bronchogenic cysts are commonly seen at the bifurcation of the trachea, followed by the posterior mediastinum and chest wall.

The mechanism of bronchogenic cysts developing in a retroperitoneal location is uncertain. One theory is that they leave the diaphragm to invade the abdominal cavity, ending in an intraperitoneal or retroperitoneal location (6). In this way, they can be seen in locations such as the liver and the adrenal gland.

The process of epithelial-mesenchymal transformation, which is important in embryogenesis, allows epithelial cells to become mobile and move through the extracellular matrix. We wonder whether this process could be the underlying mechanism underlying the development of bronchial cysts in the retroperitoneum. Is the retroperitoneal mesenchyme important in the induction of such ectopic bronchial-type tissue?

### Clinical background of adrenal incidentalomas

AI are clinically inapparent adrenal masses discovered during the investigation or treatment of conditions not related to the suspicion of adrenal disease (7). Most AI are benign, non-functioning adrenal cortical adenomas, accounting for approximately 70% of cases (8). However, a significant subset can be functional, such as cortisol-secreting or aldosterone-secreting adenomas and phaeochromocytomas, or malignant lesions, such as adrenocortical carcinomas (9). Other tumours, such as lymphomas, haemangiomas, and angiomyolipomas, may also be discovered as AI.

An important consideration in the evaluation of an AI is determining whether the lesion is benign and non-functioning (i.e. exclusion of a functioning cortical adenoma or phaeochromocytoma) (10). Rare conditions, such as bronchogenic cysts, should also be considered, especially if they appear atypical during investigation.

There may be a level of under-reporting due to a lack of histological diagnosis, whereby such cases may not be deemed appropriate for surgical resection.

## Conclusion

This case highlights the rare occurrence of a retroperitoneal bronchogenic cyst as an adrenal incidentaloma. Despite the initial presentation mimicking a non-functioning phaeochromocytoma, histopathological evaluation revealed the true nature of the cyst, emphasising the importance of considering rare differential diagnoses in the evaluation of adrenal masses. The rarity of retroperitoneal bronchogenic cysts also calls for more comprehensive studies to better understand their mechanism of formation and clinical behaviour.

## Declaration of interest

The authors declare that there is no conflict of interest that could be perceived as prejudicing the impartiality of the work reported.

## Funding

This research did not receive any specific grant from any funding agency in the public, commercial, or not-for-profit sector.

## Patient consent

Written informed consent for publication of their clinical details and clinical images (CT scan images and histopathology specimen slides) was obtained from the patient.

## Author contribution statement

TT collected the data, drafted and revised the paper, and is the corresponding author. NJ examined the specimen samples and assisted in the histopathological reporting, data collection, drafted, and revised the paper. LS interpreted the CT scan images. SR was the physician who saw the patient in clinic. DMB was the surgeon who saw the patient in the pre-operative assessment clinic. MR was the operating surgeon. RA initiated and supervised the collaborative project and revised the paper. RB examined the specimen samples, assisted in histopathological reporting, and revised the paper.
